# Hospital and Institutional News

**Published:** 1917-03-17

**Authors:** 


### HOSPITAL AND INSTITUTIONAL NEWS.

### THE WESTMINSTER HOSPITAL FIRE.

On March 7 the Westminster Hospital underwent the always exciting, but in this case fortunately not disastrous, experience of an outbreak of fire. The fire broke out about 8.30 p.m. in one of the women's wards ("Chadwick"), and is attributed to the fusing of some electric wires in the wall, near the ceiling. It is stated that a ward sister was the first to notice the flames and to give the alarm. The system of spreading the warning acted well, and in a very short space of time everyone in the building was aware of the outbreak and taking the pre-arranged measures of precaution. An eye-witness told a reporter that the doctors and nurses went to their stations as calmly and promptly as at a fire practice. The patients were removed rapidly to another ward. Some of the medical staff actually played water on the fire from a hose, and others brought stretchers for the removal of the patients. A large number of wounded soldiers are under treatment in the hospital, but it was not necessary to remove them. In a quarter of an hour from the time the fire broke out it was extinguished. The wall was damaged and the ceiling was burnt through in one corner; no other damage was done. In short, the immediate recognition of the fire, the coolness displayed by the nurses and doctors, and the smooth working of the fire-drill and precautions prevented what might otherwise have proved a disaster of real magnitude. The lesson for all institutional managers is to make sure that their arrangements would work equally well if the need were ever to arise.

### PLAIN SPEAKING BY THE LORD MAYOR OF MANCHESTER.

At the annual meeting of the Manchester and Salford Hospital Saturday and Convalescent Homes Fund it was reported that a total of £8,335 was raised in 1916, the largest sum ever obtained in one year; in 1915 £7,012 was received, and in 1914 £6,782. Of the total sum, over £5,000 was given in weekly contributions for convalescent homes, and nearly £2,000 at the annual appeal in May in works, warehouses, and offices. The Lord Mayor (Alderman Smethurst) said that although the sum raised must be considered very satisfactory in the circumstances, Manchester and Salford ought to do quite as well, if not better, than Birmingham and Leeds, which collected annually £20,000 and £18,000 respectively. Manchester and Salford, with twice the population of Leeds, ought to raise at least £36,000 in his lordship's opinion. Certainly there is something amiss when £8,000 is hailed as a record collection. What strikes the onlooker most forcibly is the small amount obtained in the works and offices; better organisation of this branch of the scheme should surely result in a larger collection than £2,000 in a city of the size and affluence of Manchester.

### COMPULSORY MEDICAL EXAMINATION.

There is a conviction held by a certain number of people that a compulsory medical examination involves something degrading to the examinee. That idea was combated a good many years ago in this journal, at a time when London Labour leaders took it into their heads to oppose the periodical medical examination of tram-drivers on the ground that such examination was "degrading to the manhood" of the said drivers. Somewhat to our astonishment, so eminently sensible and practical a person as Mr. Cecil Chapman (the stipendiary magistrate) has recently promulgated the same idea in a letter to the *Times* on the subject of the Criminal Law Amendment Bill. He "suggests" that "the degradation of mind and spirit involved in compulsory and possibly forcible examination far exceeds any benefit which is likely to accrue to the body of a person so treated, and nobody ought to be submitted to it." To which it must be rejoined, first, that Mr. Chapman's proposal to compare, to weigh in the balance as it were, moral and physical benefits is as impracticable as to compare sheep with sunsets. Secondly, that Mr. Chapman is insufficiently instructed in modern medicine and surgery to understand how great the benefit of expert treatment of venereal disease is, and how it depends upon accurate diagnosis based on laboratory findings. Thirdly, that compulsory medical examination is less terrible in its moral effects than Mr. Chapman believes: inasmuch as every recruit (and officer) admitted to the Army necessarily undergoes it. Probably Mr. Chapman's life is insured, and probably he underwent a voluntary medical examination when he proposed the contract to the insurance company. He can scarcely contend that such medical examination is degrading; and if he holds that the element of compulsion renders it so, it can only be rejoined that the protection of the public and of future generations from venereal diseases depends mainly on the effective treatment of existing cases; and that this treatment is in the last resort impossible without compulsion. *Salus populi suprema lex*, and even if we believed Mr. Chapman's views about degradation were well founded, we should still support compulsion in this matter.

### TWO NOTABLE VICTIMS OF THE WAR.

During the past week the deaths have taken place of two workers who have distinguished themselves above the ordinary amongst those who have devoted their labours to the alleviation of the lot of the wounded in war. First, the death of Mrs. Harley closes a career of energy and distinction such as few women have had the opportunity of achieving before this war. Soon after the beginning of the campaign she went to France as Commandant of the first hospital unit sent by the Scottish Federation of Suffrage Societies to Royaumont. Then she became administrator of the second similar unit, sent to Troyes by Girton and Newnham. Later on the French authorities asked the same unit to go to Salonika, and Mrs. Harley went in charge. Stationed at first at Ghevgeli, in Serbia, the unit soon had to share the retreat of the Serbian armies, and was established at Salonika. In 1916 Mrs. Harley came home and took out a motor-ambulance convoy, at first for the Scottish Women's Hospital, and later for the refugee Serbians at Monastir, where she was killed. Mrs. Harley had the French Croix de Guerre, and was a sister of Lord French. The other death is that of Major S. D. Rowland, a well-known research worker on the Lister Institute staff, who has died of cerebro-spinal fever whilst engaged in investigations into that disease, and may therefore justly be regarded as a martyr to duty. In previous years he had done much sound research upon plague and upon anti-typhoid vaccination; so one way and another much of his life-work has turned out of high military value. He went to France in 1914 in charge of the first mobile field bacteriological laboratory that has ever accompanied a British Army.

### DEATHS OF TWO DEVOTED INSTITUTIONAL WORKERS.

Death has just robbed two widely separated provincial hospitals of their most ardent workers and supporters. Alderman Stanfield Richardson, who has passed away at Sunderland in his seventh term as mayor, had completed a quarter of a century's splendid service as chairman of the Royal Infirmary, where he will be sorely missed. Lady Llewelyn, wife of Sir John T. D. Llewelyn, of Penllergaer, near Swansea, whose death has also occurred, was devotedly attached to the Swansea Hospital, which was her unceasing care. She was president of the Hospital Linen Guild, the Hospital Ball Committee and the Alexandra Rose Day, besides taking a close interest in the Hospital Convalescent Home. One of the wards at the hospital was endowed by Sir John and is known as the Penllergaer Ward. Lady Llewelyn's personal attention and her purse were ever at the disposal of philanthropic objects, especially those intended to benefit the suffering of the young. Her elder daughter maintains a home for children at Caswell Bay, near Swansea.

### LEGACIES AND THE WAR LOAN.

The Stockport Infirmary has invested £3,000 in the 5 per cent. War Loan, this sum being determined on the basis of the amount of legacies received during the last three years. In addition, its war service includes the resolve to increase its accommodation for military cases from forty-eight to fifty-eight beds—that is, to almost half the number of beds in the institution. The method adopted is to transform the soldiers' day-room into a ward, and to convert the board-room into the day-room; and in view of the urgency of the demand for more beds, the committee will meet in a room in the basement! The Stockport Infirmary has also arranged to admit certain soldiers, normally domiciled in Cheshire, suffering from paraplegia and hemiplegia, and to treat cases of venereal disease under the Local Government Board scheme. It is pleasant to add in this connection that under the will of the late Mrs. James Leigh, Manor House, Stockport, the infirmary will benefit to the extent of £500, so that by the plans adopted both the living workers and the dead friends of the infirmary are doing their best for the country to-day.

### THE REVISION OF MEDICAL EXEMPTIONS.

Partly, perhaps, as a result of the extraordinary cases recently commented on in The Hospital (February 24, 1917, p. 416; and March 10, p. 452), and partly, no doubt, as a result of similar cases which have come to light in less dramatic ways there is an agitation commencing in the press for the revision of all medical exemptions on the ground that large numbers of them have been obtained "by fraud, by favour, or by influence." With this proposal there will be widespread sympathy, although it may be doubted whether such vast numbers of scandals would be unearthed as the *Evening News* appears to suppose. Still, there can be no doubt that cases such as the White City crime have been far too frequent; and it is easy to express agreement with the contention that such revisions should be made by impartial and non-local doctors. After what was said in these columns about the White City case, it is encouraging to find an editor who takes an equally bold stand, in words which are an echo of the pronouncement on p. 452 of our last issue: "when prosecutions take place it is to be hoped that all those found guilty of subordinating their country's interest to friendship, or other considerations, will be handled wth the utmost severity." To which we would add that when the consideration is monetary, both briber and bribed should be mercilessly punished.

### GLAMORGAN'S HELP FOR THE WELSH MEDICAL SCHOOL.

The Glamorgan Finance Committee has agreed to authorise the representatives of the Glamorgan County Council selected to give evidence before the Royal Commisson on University Education in Wales to state that the old County Offices in Westgate Street, Cardiff, will be placed at the disposal of the Welsh Medical School for the immediate establishment of a National School of Preventive Medicine and Public Health Laboratory. The offer is conditional upon Treasury support equal to the local support, to continue for five years or for such time as the Treasury may lay down. It is also suggested that the grant of £1,000 a year hitherto paid to the College shall be applied to this object, together with the county's share of the salaries now paid in the Cardiff and County Joint Laboratory. It was also agreed, subject to the consent of Sir W. J. Thomas, which has now been given, that the sum of £2,000 promised by the Glamorgan County Council towards the building of the Public Health Laboratory be spent in equipping the Westgate Street premises, such equipment to belong to the County Council and to be capable of being transferred to the new laboratory erected by Sir Wm. Thomas in the Welsh Medical School. The whole proposal seems practical, sound, and generous; and a step in the right direction towards the creation of the great school of medicine which will one day spring up in Wales.

### TRIBUNALS AND HOSPITAL STAFFS.

Alderman Hampson, who lately criticised the staffing of the convalescent hospital at a meeting of the Blackpool Tribunal, withdrew his charges at the following meeting of that body. He remarked with engaging frankness that when he read his previous statements in print they went further than he intended. He had meant to refer to the statements made to him concerning the staff when exemptions were applied for by the men working there, but Colonel Barron had proved to him that the institution was actually understaffed. It possessed only one or two Class A men, who were now awaiting orders, and its Class B men were already at the disposal of the military. They were men specially fitted for their work, and therefore he withdrew his criticisms. He asked, however, if the business men in other towns were treated as strictly by other tribunals. It was only fair that an inquiry should be made. Mr. R. H. Birket, assistant military representative, said he would do his best to get the information. Hospital staffs are skilled staffs, and there would be less confusion than there is if the organisers of the National Service scheme had learnt Mr. Hampson's lesson, and instead of appealing for men in the mass had appealed for the number of skilled men in every branch that showed a deficiency. The muddle into which our agriculture has been thrown by the haphazard raids on the farmers is only one instance of the folly of not asking, and perhaps not knowing, what men are wanted, and where.

### THE EXCLUSION OF WOMEN FROM HOSPITAL BOARDS.

It is unusual for women nowadays to claim no share in hospital administration, but such appears to be the case at the Royal Sussex County Hospital, if we may judge from the statements made by Miss Blanche Fair in the local newspapers. She asserts that women are "without any voice in the management," that "the whole governing body is male," and that when information is sought by a woman "a meeting composed entirely of men thinks it compatible with right to ignore" it. This last, which is a question of manners between the person in search of information and the committee, needs no comment. The alleged exclusion of women from the management requires explanation. If true, we appeal to Mr. Ball Dodson, the chairman, in fairness to make the case plain.

### LEICESTERSHIRE'S WORK FOR WAR CHARITIES.

A magnificent record of patriotic work was presented at the annual meeting of the Alexandra Collections Committee, presided over by the Mayor of Leicester. The hon. treasurer (Alderman Tollington, J.P.) submitted the audited accounts, which showed that the total sums raised during the year for the British and Allied societies amounted to £37,750, an average collection of £722 per week. Nine efforts in the county area resulted in contributions of £2,476. The total sums raised in the first two years of war amounted to just under £50,000. Mr. E. C. Kemp, the hon. secretary, reported that the cost of the collection of this large sum was approximately 4¼ per cent., which he thought would be found to compare very favourably with any similar organisation. In recognition of the contribution of £2,382 raised for the Russian Red Cross, the Empress of Russia had expressed her desire to confer the Imperial Russian Order upon the Mayor and Mayoress, and it is understood that other members of the Committee are also likely to receive decorations. The Empress has desired that the coat of arms of the borough should be carved in wood and forwarded to the War Hospital in Petrograd, where it will be hung over the beds provided by the town's generosity. The success of the organisation has been due to the enthusiasm and devotion of a band of Alexandra Ladies, numbering over 1,100, known as the Mayoress's Grand Council, who, sometimes under most trying climatic circumstances, have heroically carried on their work without a murmur of discontent. Not a single penny has been paid in salaries. The meeting decided to organise flag days in the current year for the Leicester Y.M.C.A. War Hut Fund, the Leicestershire Prisoners of War Fund, the Buckingham V.C. Memorial for providing scholarships for the children of the Countesthorpe Cottage Homes under the direction of the Leicester Board of Guardians, the Mayor's Fund for Disabled Soldiers and Sailors, the Leicester War Hospitals Games Committee, Alexandra Day for the local charities, and the National Sailors' Day and other worthy objects not yet decided upon. The officers and committees of the Society were re-elected, with cordial expressions of appreciation of the splendid work accomplished.

### MR. HARGREAVES IMPROVES THE OCCASION.

The art of improving the occasion has been ably used by Mr. F. A. Hargreaves on completing twenty-one years of work as honorary secretary to the Victoria Hospital, Burnley. At the recent annual meeting he gave a succinct and well-arranged sketch of the institution's history, and delighted his listeners with a summary of the work and personalities of those who had made its growth their chief care from its inception in 1882 to the present day. The early efforts at collections in the mills and the first subscriptions were summarised, as well as the result of the competition for the building, which was judged by Captain Douglas Galton, and the prize awarded to a Burnley firm. This energy has lasted throughout, and the extension fund, begun in 1911, stands to-day at £34,063. It has had, too, its influence on the staff, and it is noteworthy that the hospital has had only three matrons since Miss Piggott's day. The present matron, Miss Lindsay, has held the post for over eighteen years. The art of making history live is not one to be neglected by hospital managers, and Mr. Hargreaves could not have done better than by his arresting address to foster the tradition which has accomplished so much already for his hospital.

### AN INTERESTING ANNIVERSARY.

Two interesting points were associated with the annual meeting of the Montgomery County Infirmary on the 1st instant. One was that the meeting was held on St. David's Day, the anniversary of the foundation of the infirmary in 1868, when the original building was opened with eight beds. The other point was of personal interest, as the annual meeting was the twenty-fifth attended in an official capacity by Mr. Ernest C. Morgan, the secretary. The Montgomery Infirmary has grown since 1868; the present buildings, erected in 1911, contain forty beds, and it is hoped shortly to provide a further fourteen beds. The institution is taking its share of wounded soldiers, and during several months of last year two large wards were given over for their accommodation. The income and expenditure account showed a balance to the good of £173. The only criticism of the report that we have to offer is that this progressive county infirmary should present its accounts under the Uniform System.

### THE END OF A SORE POINT IN WALES?

The accounts of the Welsh National Memorial Association have been so much under discussion of late that there is an added interest in comments thereon which appear in the White Paper on the work of the National Insurance Audit Department issued last week. "The auditor," says the White Paper, "directed attention to certain adjustments necessary in the accounts (of the Memorial Association) to disclose the proper incidence of the burden of the net cost of the scheme. Adjusted accounts for these two years were accordingly presented for audit in the period under review. In his report on these adjusted accounts the auditor directed attention to various defects, all of which appear to have been considered by the Association, and in many cases to have been dealt with in an appropriate manner. In particular, there has been an improvement in the form and method of keeping the accounts, inventories of furniture and other effects on the Association's several properties are in course of preparation, and the title deeds required for inspection by the auditor have now been produced." This explanation, one hopes, may be allowed to close a chapter of criticism which undoubtedly has left on the public mind a prejudicial impression of the accountancy methods of the Welsh National Memorial Association. The improvements effected should restore public confidence in the Association's capacity to administer with wise efficiency the money contributed by the constituent councils and public bodies.

### THEFTS OF HOSPITAL STORES.

Thefts of hospital stores—some of them on rather an extensive scale—have been on the increase of late, and the facts in certain of the cases, as elicited in the police courts, seem to call for greater watchfulness over the supplies department. In one of the cases the *chef*—an Italian—at a military hospital in London was sent to prison for a month in the second division for stealing comparatively small quantities of meat, bread, and flour. It was stated that his wages were three guineas a week, and that he had his meals at the hospital. Another case came from a military hospital at Aberdeen; the stolen stores in this instance included 17 lb. of tea, 560 lb. of sugar, 320 lb. of oatmeal, 10 lb. of cheese, besides other goods. The chief offender here was sentenced to three months, the other defendant —a university graduate, and formerly a master in one of the largest schools in the city—was fined £15. At a Lancashire court a penalty of £5 was imposed on a greengrocer for being in possession of 28 lb. of margarine issued for use at a military hospital. Policemen who had secreted themselves saw the defendant in this case drive into the hospital grounds, lift an apparently empty hamper off his cart, and go into the cook-house. Half an hour later the man came out with the hamper, now very heavy and covered with a rug. On examination there was found in the hamper tins of condensed milk, fish, hams, beef, meat extract, and a box of margarine, the latter being labelled "Military supplies."

### ARE SOLDIERS' COMFORTS TO BE CURTAILED?

Over one hundred soldiers are being accommodated at the Bradley Gate Sanatorium, Huddersfield, which was taken over this month as a military hospital. The site of fourteen acres, which was given by the late Mr. John Sykes to the Corporation first for a children's sanatorium, to which wards for adults have since been added, is a good one. The trustees have consented to the use of the children's wards as well. In accepting the hospital on behalf of the War Office, Surgeon-General W. G. Bedford said, "As for the wounded, all provision which was possible must be made within reasonable limits." In the present circumstances they could "not expect that same high standard of luxury and comfort which, perhaps, in the early days of the war the public normally looked upon as the bounden right of the wounded." Perhaps Surgeon-General Bedford will explain what he means by this dubious statement, for if the wounded are not to have that "high standard of luxury and comfort which the public look upon as their bounden right," it is important to know what luxuries and comforts are to be curtailed. We trust he was referring merely to those luxuries which are actually detrimental to the health of the soldiers, such as excess of alcohol or of tobacco.

### ON TAKING RISKS IN PHILANTHROPY.

Is the chairman of a hospital justified in taking risks in his policy or undertakings on behalf of the institution for which he labours? Some chairmen do so, as in a published advertisement which we described as "humorous?" but which one of our correspondents says struck him as merely preposterous. An advertisement, continues this correspondent, which may astonish, or baffle, or give rise to possible offence, is neither more nor less than taking an unnecessary risk. It is conceivable that it made enemies in ranks where friendship ought to be sought. Yes, all said and done, it was a risk. And there are other risks in philanthropy that are taken. A chairman may preside over a committee that takes the risk of over-building; he may favour the engaging of a member of the medical or administrative staff who is known to be a man of dangerous views or prejudices; he may make a big mistake, and afterwards take the risk of attempting to justify it.

### CHARITY AND SPECULATIVE ENTERPRISE.

Yet it is a matter of opinion as to whether it is not sometimes necessary to take risks in philanthropy, for there are some circles which are so steeped in tradition and conservatism that it is not easy to move them, unless the risk is taken of surprising them into action by a speculative enterprise. Some of the most successful schemes in the philanthropic world have been the result of taking risks. Timidity and conventionality may have had their day. We are now living in new times. Enterprise in philanthropic affairs, as in national affairs, is not to be despised. The man who runs a shop, or an office, or a hospital must keep his eyes open if he would stay at the top.

### A NEW USE FOR LEGACIES.

The recent gift of £10,000 from Mr. Charles Lyle, through Mr. Leo Lyle, chairman of the Queen Mary's Hospital for the East End, marks an era in the history of the institution recently known as the West Ham and Eastern General Hospital. In these days, when the East End is liable to suffer from fire or bombs, those who own factories in the district might hesitate to give large sums, even though their works are doubtless covered by insurance. But it says much for the precautions taken and the financial foresight displayed that there has been little, if any, weakening of local generosity in London. We may revert to the subject, since apparently no conditions have been imposed, and the building of a maternity home or even a throat and ear hospital have been suggested. Before a decision is arrived at, suggestions should be welcome; and, therefore, we think that the desirability of considering an increase in the salaries of the staff should be considered. The point is not that new buildings may not be wanted, but that someone must set an example of remedying a defect in existing hospital administration—namely, the notorious underpayment of hospital staffs. New buildings are fairly safe of securing support. The pinch of their absence is publicly felt. But hospital staffs have no one to plead for them but themselves. Why should not some hospital or donor set an example by devoting unconditional legacies to this purpose?

### THE WELCH FAMILY AND THE LANCASHIRE INFIRMARY.

The appointment of Mr. W. G. Welch as president of the Royal Lancashire Infirmary marks an interesting stage in a long record of family service to hospitals. Nearly fifty years have passed since he became a subscriber. In 1879 he was appointed auditor, and has sat on the committee after retiring from this post. Mr. Welch's brother, Mr. A. B. S. Welch, has just completed twenty-five years' service, and as honorary auditor his loss will be felt, since his resignation has been due to the claims of other work upon his time and energies. To build up family connections is the surest and happiest part of hospital administration, in which, indeed, the whole district served by a hospital should feel a family interest.

### THE REWARD OF SKILL AND SERVICE.

The work of an honorary secretary to a cottage hospital entails all the time and trouble of a responsible post, but is usually left to the scant leisure of an already busy man. It is therefore always agreeable to note public recognition of long service in these offices, and we welcome the tribute paid to Mr. F. R. Britton after his retirement from the honorary secretaryship of the Abergavenny Cottage Hospital. He had held the post for eleven years, and the regard in which he is held has been marked by appointing him as a vice-president for life and giving him a seat on the board of management. Mr. A. M. Cunliffe has succeeded him. An appreciative board has granted the matron a gratuity of £10 for her services and the economies she made at a difficult time.

### A GOLD COLLECTION.

As a result of the belief that a considerable amount of hoarding still goes on in obscure corners of the country, Mr. Eastlake Smith has suggested that a double purpose might be served, and the Sydenham Hospital for Sick Children be benefited, if a gold collection were held in the district. It sounds a desperate remedy, and we should like to hear from Mr. F. H. Carter, the honorary treasurer of the hospital, what success is gained or expected. The needs of a hospital move many hearts, and the fact that gold is hoarded by naturally close-fisted people is no necessary argument against this novel scheme.

### ECONOMICAL MANAGEMENT IN CORNWALL.

A record of careful supervision and efficient management that achieved quite extraordinary results in these days of all-round dearness was unfolded at the annual meeting of the West Cornwall Miners' and Women's Hospital, one of the oldest of Redruth's institutions. The food bill for the year amounted to £928; the average cost per head (including staff) worked out at £17 8s. 8d., or 6s. 8d. a week, compared with £18 8s. 11d., or 7s. 1d. per head per week in the previous year. The total cost per bed per patient per annum also showed a saving—£71 1s. 11d., against £78 2s. 4d. The committee, commenting in their report upon this achievement, said that, "thanks largely to the efforts of the matron and staff," the greatly increased price of everything had only involved a small debit balance of £2 1s. The Chairman, Mr. James Wickett, pointed out that the institution, with an increased number of patients, had been run with a small decrease in cost. That result, he said, could only have been brought about by admirable management on the part of the committee, matron, and staff. More money was, however, required, and he should lead the way with his subscription, for he was more interested in the hospital than ever. Viscount Clifden was re-elected president of the institution.

### RATE RELIEF FOR WAR CHARITIES.

Although our hospitals and old-established charities have sometimes met with lenient treatment in the matter of assessment for local rates, none, within our memory, have got off scot free. Whether by recent action on the part of local authorities a precedent has been set up which may lead to preferential treatment in this respect it is hard to say, but it is a definite fact that some of the war charities have succeeded in freeing themselves from the demands of the local rate-collector. This concession must be considered a temporary thing applied to temporary bodies, and appears to be granted only in cases where no rent is paid; thus the fortunate war charity that gets free accommodation has also freedom from rates. Truly there is Biblical authority for such treatment, but this will bring small comfort to the war charity which has not found a generous landlord. The Local Government Board has promised not to raise the question of the legality of the proposed arrangement, which has been undertaken by the Holborn Borough Council and the Councils of a number of Metropolitan Boroughs.

### A "ROLL OF HONOUR" HOSPITAL.

The institution in the Harrow Road hitherto known as the Women's Hospital for Children, which is managed and staffed entirely by women, is to change its name to "The Roll of Honour Hospital for Children," because it is hoped that when a contemplated building scheme becomes practicable after the war, the whole of the cots will be endowed in memory of men who have fallen in the present war. The idea of obtaining hospital endowments to commemorate the fallen is a very good one, and has been more than once eulogised in these columns; but for a new-comer whilst in makeshift buildings, to ask the public to endow every bed is not an action we can support for clear reasons. The proposed title seems cumbrous, though it would be difficult to suggest a shorter one which conveys the basic idea of this endowment scheme. If after the war this institution continues and an endowment scheme is put forward with success, there will be nothing more to be said about it. It will be within the recollection of our readers that in The Hospital of January 31, 1914, p. 481, we described the existing buildings of this hospital, and criticised the policy of attempting to care for in-patients in the rather ramshackle tenements in which it is housed. Should the new building now proposed be erected after the war, the work and efficiency of this hospital ought to be material.

### THE CONDITIONAL DONATION.

Charities and those who have to do with charities are familiar with the conditional donation, another instance of which has been announced in respect of the Royal Hospital for Diseases of the Chest, City Road. In this case Mr. William Hartmann, of Esher (a gentleman whose name will be found in the subscribers' lists of many Metropolitan charities), has generously offered to contribute £1,000 to the hospital, conditionally upon a further seven subscriptions, each of a similar amount, being received before March 31 next. There is a sporting element about these offers which most often leads to success; in fact, the failures, if failures ever happen, are never put on record. We should imagine that those who have liberally supported the hospital in the past, when the conditional donation is announced, know what to expect and make up their minds to prepare a response to a heartrending appeal to save the situation. We wonder what would happen if only five other thousands are offered; will the conditional donor be so hard-hearted as to stick to the letter of the bargain, and would the five other conditional donors he has conjured into being support him in his act of ruthlessness?

### THIS WEEK'S DRUG MARKET.

As was not unexpected, English refiners of camphor have again advanced their prices, but even the new quotations are several pence per pound below the prices asked for Japanese refined; taking all the circumstances into consideration, it is not improbable that English refiners will advance their prices still further in due course. Borax and boric acid are dearer. Very high prices are being paid for honey, and it is probable that the highest figure has not yet been reached. Carthagena ipecacuanha is scarce and worth as much as, or even rather more than, Mattogrosso ipecacuanha. Citric acid, tartaric acid, and cream of tartar continue to tend higher in price. For glycerophosphates high prices have to be paid, and it seems certain that the value will advance still further in consequence of the shortage of these salts due to the restrictions on glycerine and phosphoric acid. A fair business is being done in quinine at well-maintained prices. Sugar of milk is again rather dearer. Myrrh continues to be scarce. Any gum asafœtida available fetches very high prices. Balsam copaiba has again advanced in price owing to scarcity. Gum ammoniacum is badly wanted, but more appears to be offered. Balsam Tolu is scarce and dearer. In synthetic drugs there are few price-changes of importance, but the downward tendency in the value of phenacetin continues. It remains to be seen what will be the effect (on the prices here of synthetic drugs and other so-called "fine chemicals" used in medicines) of the prohibition on the exports of these products from Switzerland.

